# Characterization of Copy-Number Variations and Possible Candidate Genes in Recurrent Pregnancy Losses

**DOI:** 10.3390/genes12020141

**Published:** 2021-01-22

**Authors:** Yan-Ran Sheng, Shun-Yu Hou, Wen-Ting Hu, Chun-Yan Wei, Yu-Kai Liu, Yu-Yin Liu, Lu Jiang, Jing-Jing Xiang, Xiao-Xi Sun, Cai-Xia Lei, Hui-Ling Wang, Xiao-Yong Zhu

**Affiliations:** 1 Laboratory for Reproductive Immunology, Hospital of Obstetrics and Gynecology, Fudan University, Shanghai 200000, China; 18111250011@fudan.edu.cn (Y.-R.S.); hwt224@163.com (W.-T.H.); weichunyan90@gmail.com (C.-Y.W.); 15211250008@fudan.edu.cn (Y.-K.L.); 172112500112@fudan.edu.cn (Y.-Y.L.); 2 The Affiliated Suzhou Hospital of Nanjing Medical University, Suzhou Municipal Hospital, Suzhou 215000, China; houshunyu@sina.com (S.-Y.H.); jianglubeijia@163.com (L.J.); Xiangjingjing2013@163.com (J.-J.X.); 3 Shanghai Ji Ai Genetics & IVF Institute, Obstetrics & Gynecology Hospital, Fudan University, Shanghai 200000, China; jiai_sh@163.com (X.-X.S.); xiaoxi_sun@aliyun.com (C.-X.L.); 4 Key Laboratory of Reproduction Regulation of NPFPC, SIPPR, IRD, Fudan University, Shanghai 200000, China; 5 Shanghai Key Laboratory of Female Reproductive Endocrine Related Diseases, Shanghai 200000, China

**Keywords:** copy-number variations, sporadic abortion, recurrent pregnancy losses, genetic etiology

## Abstract

It is well established that embryonic chromosomal abnormalities (both in the number of chromosomes and the structure) account for 50% of early pregnancy losses. However, little is known regarding the potential differences in the incidence and distribution of chromosomal abnormalities between patients with sporadic abortion (SA) and recurrent pregnancy loss (RPL), let alone the role of submicroscopic copy-number variations (CNVs) in these cases. The aim of the present study was to systematically evaluate the role of embryonic chromosomal abnormalities and CNVs in the etiology of RPL compared with SA. Over a 3-year period, 1556 fresh products of conception (POCs) from miscarriage specimens were investigated using single nucleotide polymorphism array (SNP-array) and CNV sequencing (CNV-seq) in this study, along with further functional enrichment analysis. Chromosomal abnormalities were identified in 57.52% (895/1556) of all cases. Comparisons of the incidence and distributions of chromosomal abnormalities within the SA group and RPL group and within the different age groups were performed. Moreover, 346 CNVs in 173 cases were identified, including 272 duplications, 2 deletions and 72 duplications along with deletions. Duplications in 16q24.3 and 16p13.3 were significantly more frequent in RPL cases, and thereby considered to be associated with RPL. There were 213 genes and 131 signaling pathways identified as potential RPL candidate genes and signaling pathways, respectively, which were centered primarily on six functional categories. The results of the present study may improve our understanding of the etiologies of RPL and assist in the establishment of a population-based diagnostic panel of genetic markers for screening RPL amongst Chinese women.

## 1. Introduction

Spontaneous abortion is the most common complication of pregnancy in women of childbearing age, accounting for about 15% of clinical pregnancy cases during the first trimester [1]. Excluding sporadic miscarriage (one natural abortion history, sporadic abortion; SA), recurrent pregnancy loss (RPL) is defined by the European Society for Human Reproduction and Embryology (ESHRE) November 2017 guidelines as the loss of two or more pregnancies [2], and recurrent miscarriage (RM) by the Royal College of Obstetricians and Gynecologists (RCOG) as at least three consecutive miscarriages before 24 weeks of gestation [3]. The incidence of the latter is 5% and has shown to be exhibiting an increasing trend in recent years [4]. The repeated loss of pregnancy is physically, mentally, and emotionally challenging for both doctors and couples trying to conceive, especially on the mother.

As a complex polygenic and multifactorial disease, there are multiple etiologies underlying RPL, but 50% of cases can be attributed to genetics, such as aneuploidy. Additionally, a spectrum of non-genetic etiologies of RPL have also been identified, including maternal thrombophilic disorders, uterine abnormalities, sperm DNA fragmentation, immune and endocrine disturbances, and even lifestyle factors such as drinking and smoking [5,6]. Numerous studies have been performed to determine potentially genetic markers associated with pregnancy loss, more recently taking advantage of high-resolution molecular techniques, including chromosomal microarray analysis and next-generation sequencing. However, the results vary from study to study. Although large copy-number variations (CNVs) are well known to be associated with the risk of miscarriage [7], specific information based on large and systematic cohorts regarding the relationship between specific CNVs and RPL is limited. Moreover, it is unclear whether there exists a distinction of CNVs between cases of SA and cases of RPL.

To elucidate the potential differences in the incidence and distribution of chromosomal abnormalities between SA cases and RPL cases systematically, and to identify new specific CNVs likely to be involved in the etiology of RPL, single nucleotide polymorphism array (SNP-array) and CNV-sequencing (CNV-seq) were performed in more than 1500 cases of miscarriages in the present three-year retrospective study. Collectively, we analyzed the critical regions of the detected CNVs to identify potential RPL candidate genes and further functional gene analysis using gene enrichment and protein interaction analysis. The results of the present study may contribute to the establishment of a population-based diagnostic panel of genetic markers for RPL screening amongst Chinese women.

## 2. Materials and Methods 

### 2.1. Study Subjects

**Figure 1:**
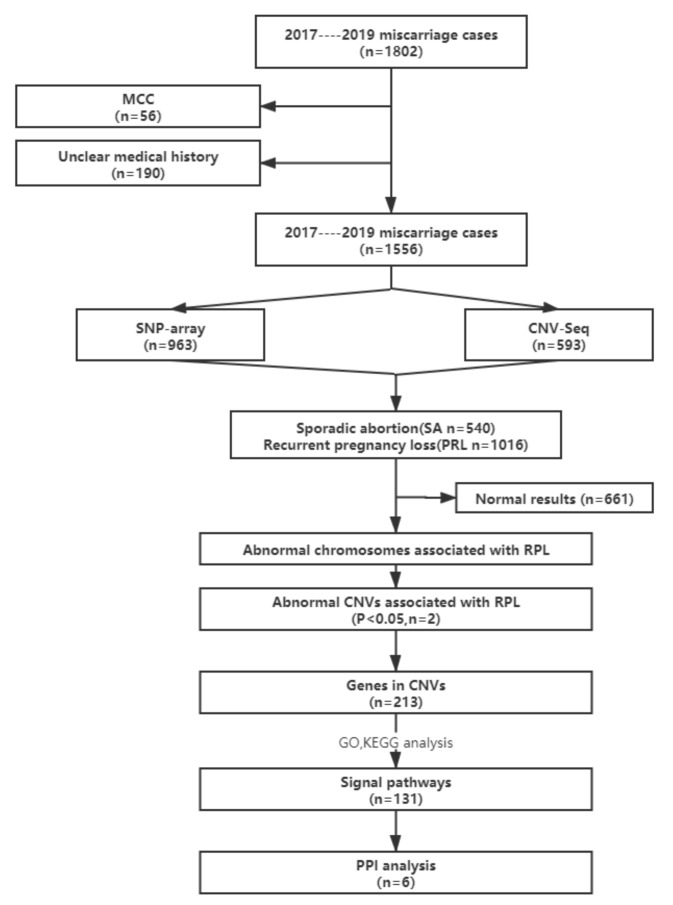
Flow diagram of the cases of miscarriage included, and the analytical strategies used in the present study. MCC, maternal cell contamination; SNP-array, single nucleotide polymorphism array; CNV-seq, copy number variation sequencing; RPL, recurrent pregnancy loss; PPI analysis, protein-protein interaction analysis.

This retrospective study was approved by the Institutional Ethics Committee of Shanghai JIAI Genetics and IVF Institute, Obstetrics & Gynecology Hospital of Fudan University in mainland China between 2017 and 2019, and written informed consent was obtained from all participants. A total of 1802 cases of miscarriage were referred to Shanghai JIAI Genetics and IVF Institute for diagnosis and treatment. Fresh products of conception (POCs) were collected according to standard clinical procedures. Genomic DNA was extracted from all POCs using a QIAamp DNA Mini Kit (Qiagen GmbH, Hilden, Germany). Samples with significant maternal cell contamination (MCC) exceeding 30% were excluded (56 samples), which was determined using short tandem repeat profiling, as described previously [8]. Additionally, 190 cases with an unclear medical history were also excluded from the study. Since maternal thrombophilic disorders are the second most frequent cause of RPL, patients with thrombophilic conditions were excluded. Therefore, a total of 1556 miscarriage cases were included in the current study. Analysis of chromosomal abnormalities and CNVs was performed using SNP-array or CNV-seq. The number of cases of miscarriage and analytical strategies are summarized in Figure 1.

### 2.2. SNP-Array Analysis

The HumanCytoSNP-12 DNA Analysis Bead Chip (Illumina, Inc., San Diego, CA, USA) was used for SNP-array analysis according to the manufacturer’s protocol and molecular karyotype analysis was performed using KaryoStudio V 1.4.3.0 (Illumina, Inc., San Diego, CA, USA). A range of chromosomal abnormalities were included: (1) autosomal and sex chromosome aneuploidy; (2) mosaicism for autosomal and sex chromosome aneuploidy greater than 30%; (3) deletion of CNVs on chromosomes greater than 1 mega base pair (Mb); (4) CNV duplications on chromosomes greater than 2 Mb; (5) loss of heterozygosity greater than 5 Mb; (6) mosaicism for deletion, duplication of CNVs ≥ 5 Mb greater than 30%.

### 2.3. CNV-seq

CNV-seq was performed as reported previously, with minor modifications [9]. Briefly, 50–100 ng genomic DNA was fragmented and used for construction of DNA libraries through adapter ligation and PCR amplification. An Ion Proton Sequencer (Thermo Fisher Scientific, Inc., Waltham, MA, USA) was used to sequence DNA libraries to generate about 4–5 million raw single-end sequencing reads of approximately 200 base pairs in length. There were a total of 2.8–3.2 million uniquely mapped reads aligned to the University of California Santa Cruz (UCSC) Human Genome Build 19 (hg19) (Genome Reference Consortium (GRC) Build 37) using the Burrows–Wheeler algorithm [10] and allocated to a 20-kilobase (kb) bin on each chromosome. CNVs were identified using a circular binary segmentation algorithm [11]. A three-step GC correction, including LOESS regression, intrarun normalization and linear model regression, was performed to eliminate GC bias between different samples as described previously [12].

### 2.4. Evaluation of CNVs

Databases (ISCA, DGV, Decipher, Ensemble, OMIM, ClinGen, UCSC and PubMed) were used to analyze the suspected pathogenic regions. The pathogenicity of CNV regions detected was primarily determined from these databases and empirical diagnosis. The presumed pathogenicity or non-pathogenicity of the detection results were only considered indicative and confirmatory.

### 2.5. Statistical Analysis

A χ^2^ or Fisher’s exact test was used to compare the frequency of CNVs between the SA cases and the RPL cohort. *p* < 0.05 was considered to indicate a statistically significant difference. Statistical analyses were performed using SPSS software (version 22.0, IBM Corp., Armonk, NY, USA).

### 2.6. Functional Enrichment Analysis

The genes located in the assessed significant CNV regions were referred to in the UCSC genome browser (http://www.genome.ucsc.edu/). Enrichment was tested for the functional categories defined in Gene Ontology (GO) and Kyoto Encyclopedia of Genes and Genomes (KEGG). In the current study, we considered statistically significant enrichment when the adjusted *p*-value was <0.05. Significant GO results were further chosen to construct a protein–protein interaction network (PPI network) using String (https://string-db.org/). The candidate signaling pathways were chosen based on generally accepted biological mechanisms contributing to embryogenesis and development, as well as susceptibility to, development of, and progression of pregnancy-associated diseases.

## 3. Results

### 3.1. Specimen Characteristics

Initially, 1802 cases of miscarriage, where the Shanghai JIAI Genetics and IVF Institute, Obstetrics & Gynecology Hospital of Fudan University was consulted, between 2017 and 2019, were included in this study. We excluded 56 cases due to significant MCC and 190 cases with unclear medical history. Of the 1556 miscarriage cases ultimately included in this study, 963 cases were tested with SNP-array and 594 cases with CNV-seq. Of the cases, 540 (34.70%) cases were SA (SA group) and 1016 (65.30%) cases all experienced two or more miscarriages (RPL group) (Figure 1). In the SA group, no recurrence of miscarriage occurred during subsequent follow-up performed until 31 July 2020. Of the RPL patients, 586 (37.66%) cases suffered two miscarriages, 283 (18.18%) cases suffered three miscarriages and 147 (9.45%) cases suffered four or more miscarriages. The mean gestational week at the time of miscarriage was 9.5 (range, 5–14) weeks; the mean maternal age of the SA group was 30.47 (range, 21–46) years old, and in the RPL group, 32.17 (range, 21–48) years old. Of the RPL cases, the age of the patients with two, three or four or more miscarriages was 31.63 (range, 21–45) years old, 32.43 (range, 22–44) years old, and 33.95 (range, 24–48) years old, respectively (Table 1). Maternal age and number of prior miscarriages have been consistently found to be risk factors for RPL [2]. In this study, the number of miscarriages increased with age, which may be related to the increased probability of trisomy 21 and trisomy 18 in older pregnant women [13]. However, the causes of RPL are not only related to the mother’s age, but also may be related to infection, thrombotic diseases and immunity, amongst other factors [14], and attention should be paid to these factors during clinical consultation, as the majority of RPL cases occur with an unmodifiable risk factor [15].

### 3.2. Chromosomal Abnormalities Detected by Chromosomal Microarray Analysis and CNV-seq

**Figure 2:**
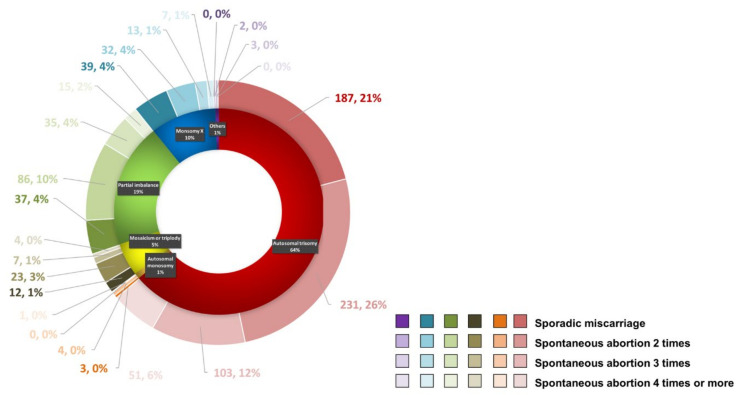
Types and frequencies of chromosomal abnormalities detected in the products of conception in cases with different times of miscarriage using single nucleotide polymorphism-arrays and copy number variation-sequencing.

As mentioned above, a total of 1556 cases with POC results were available for further analysis, including 963 cases detected using SNP-array and 593 cases tested using CNV-seq. Overall, normal results were identified in 661 (42.48%) cases and abnormal results were identified in 895 (57.52%) cases (Table 1). Of the 895 cases with abnormal results, aneuploidy was the most common abnormal finding with 572 (64%) cases of autosomal trisomy, 91 (10%) cases of monosomy X, and 8 (1%) cases of autosomal monosomy (Figure 2 and Table 2). Autosomal trisomy with simultaneous sex chromosome polyploid was included in the group of autosomal trisomy. Mosaicism or triploidy was found in 46 (5%) cases. Partial imbalance (CNVs) was observed in 173 (19%) cases. Other cases included one case of XY trisomy, one case of tetrasomy 16 with XY trisomy, one case of tetrasomy 8, one case of tetrasomy 21, and one of case polyploidy. With the increase in the number of SAs, the chromosome abnormality rate of embryos first increased, then decreased. The chromosomal abnormality rate of patients with two abortions was the highest (42%), but there was no statistical difference between the groups (*p* > 0.05). Therefore, we grouped patients with two or more miscarriages into one group, and compared them with the control group of SA.

**Figure 3:**
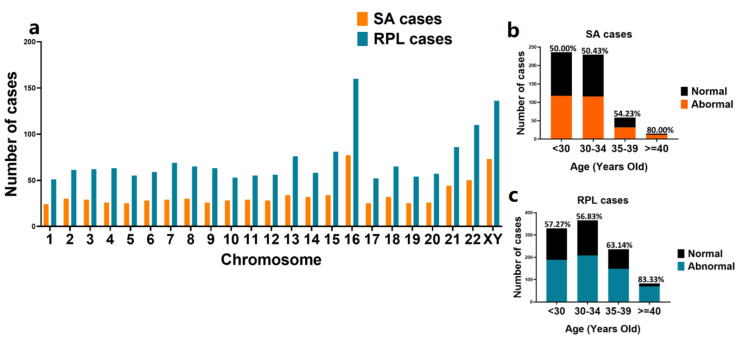
Frequency of chromosomal abnormalities detected in SA and RPL cases of different ages. (**a**). The distribution of chromosomal abnormalities in the chromosomes in the SA and RPL groups. (**b**). The incidence of chromosomal abnormalities detected in SA cases of different ages. (**c**). The incidence of chromosomal abnormalities detected in RPL cases of different ages. SA, sporadic abortion; RPL, recurrent pregnancy loss.

Abnormalities were observed in all chromosomes; the RPL group had a higher incidence of chromosomal abnormalities than the SA group, but there were no statistical differences between the two groups (Figure 3a). Abnormalities of chromosome 16 were the most frequent, consistent with a previous study [7]. XY chromosomes were the second most frequent, while chromosome 1 had the lowest incidence of abnormality. We also investigated the relationship between maternal age and chromosomal abnormalities, finding that in the SA group, the frequency of chromosomal abnormalities increased with maternal age (Figure 3b), whereas in the RPL group the frequencies of chromosomal abnormalities were the lowest in the 30–34-year-old age group (Figure 3c). This finding might support the use of preimplantation genetic testing for aneuploidy in patients who experience RPL to improve live birth rates.

### 3.3. Identification of Recurrent CNVs Associated with RPL

**Figure 4:**
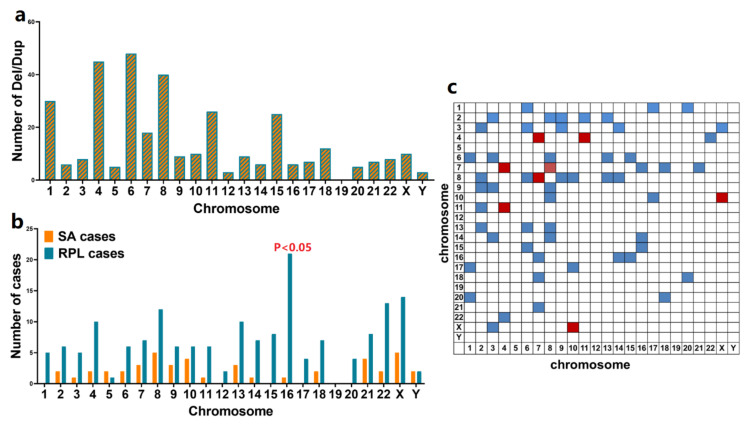
CNVs detected in POCs in using SNP-array and CNV-seq. (**a**). The number of deletions or duplications of CNVs on each chromosome. (**b**). Cases with dup/del of CNVs on two different chromosomes. CNVs in chromosomes 4 and 7, chromosomes 4 and 11, chromosomes 7 and 8, and chromosomes 10 and X were observed in 2 cases and highlighted with a red box. (**c**). The cases of CNVs detected in SA and RPL cases. CNV, copy number variation; POC, products of conception; SP, single nucleotide polymorphism; SA, sporadic abortion; RPL, recurrent pregnancy loss.

To identify significant CNVs related to RPL, a total of 346 CNVs in 173 cases were subjected to further analysis (Table S1) including 272 duplications, 2 deletions and 72 duplications along with deletions, after excluding cases with numerical chromosomal abnormalities. In these identified CNVs, only two CNVs were large CNVs (≥10 Mb), and the remaining CNVs were submicroscopic CNVs (<10 Mb). The distribution of all detected CNVs in all chromosomes is shown in Figure 4a. Except chromosome 19, CNVs were observed in all chromosomes. The two deletions occurred in 8p23.1 and 14q32.12. The duplications occurred mostly in chromosome 6, followed by chromosomes 4 and 8, but there were no statistical differences between chromosomes (*p* > 0.05). Cases with CNVs on two different chromosomes are summarized in Figure 4b. CNVs in chromosomes 4 and 7, chromosomes 4 and 11, chromosomes 7 and 8, and chromosomes 10 and X were observed in two cases and are highlighted with a red box.

More patients in the RPL group than the SA group had CNVs in their chromosomes, except for chromosomes 5 and Y (Figure 4c). That is to say, the CNV rate of RPL patients was higher than that of the SA group. No CNVs were detected on chromosome 19 in both groups. In the SA cases group, no CNVs were detected on chromosome 1, 12, 15, 17 and 20. In addition, we identified 74 recurrent (*n* ≥ 2) CNVs in the group of RPL cases. Of these CNVs, two were identified with significantly higher frequencies in RPL cases than in the SA cases group. These two statistically significant recurrent CNVs involved duplications of 16q24.3 and p13.3 and were considered to be associated with RPL.

### 3.4. Identification of RPL Candidate Signaling Pathways and Genes

**Figure 5:**
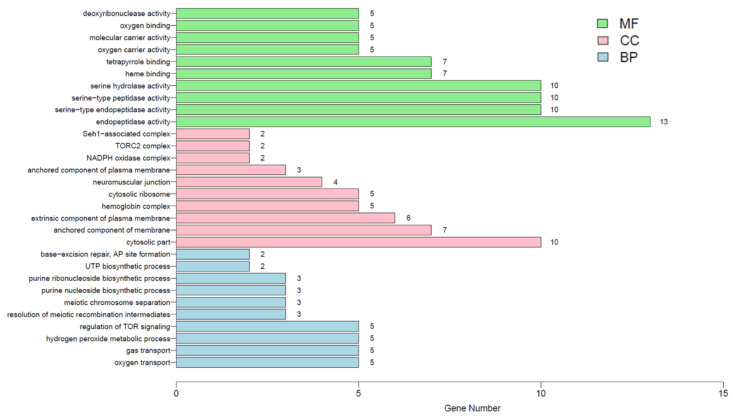
The top 10 enriched pathway results in three categories with *p* < 0.05 of Gene Ontology analysis. MF, Molecular Function; CC, Cellular Component; BP, Biological Process.

**Figure 6:**
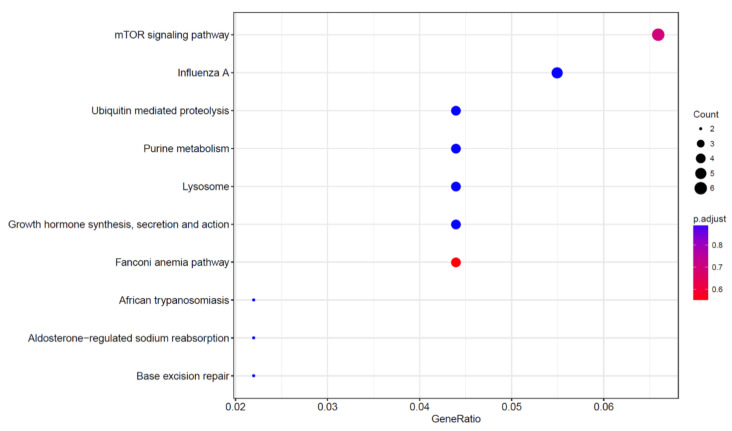
Enrichment results of analysis using the Kyoto Encyclopedia of Genes and Genomes.

To determine the critical genes and related signaling pathways associated with RPL from CNVs, the number of genes and the genomic position located in the significant recurrent CNVs (16q24.3 and p13.3) were systematically evaluated using the UCSC genome browser (http://www.genome.ucsc.edu/). All 213 genes within the two regions are summarized in Table S2. We further examined the enrichment of the 213 genes using the Gene Ontology (GO) analysis along with Kyoto Encyclopedia of Genes and Genomes (KEGG) analysis. The results of GO analysis are shown in Figure 5 and Table S3, and results of KEGG enrichment analysis are shown in Figure 6 and Table S4. GO analysis showed that the 213 genes were significantly enriched in 131 different signaling pathways (*p* < 0.05) the most common of which was “hemoglobin complex” (*p* = 1.33 × 10^−7^). KEGG analysis showed that the “Fanconi anemia pathway” (*p* = 0.003) was the most commonly enriched of the 173 signaling pathways identified, followed by the “mTOR signaling pathway” (*p* = 0.008), “Influenza A” (*p* = 0.04) and “Growth hormone synthesis, secretion and action” (*p* = 0.046).

**Figure 7:**
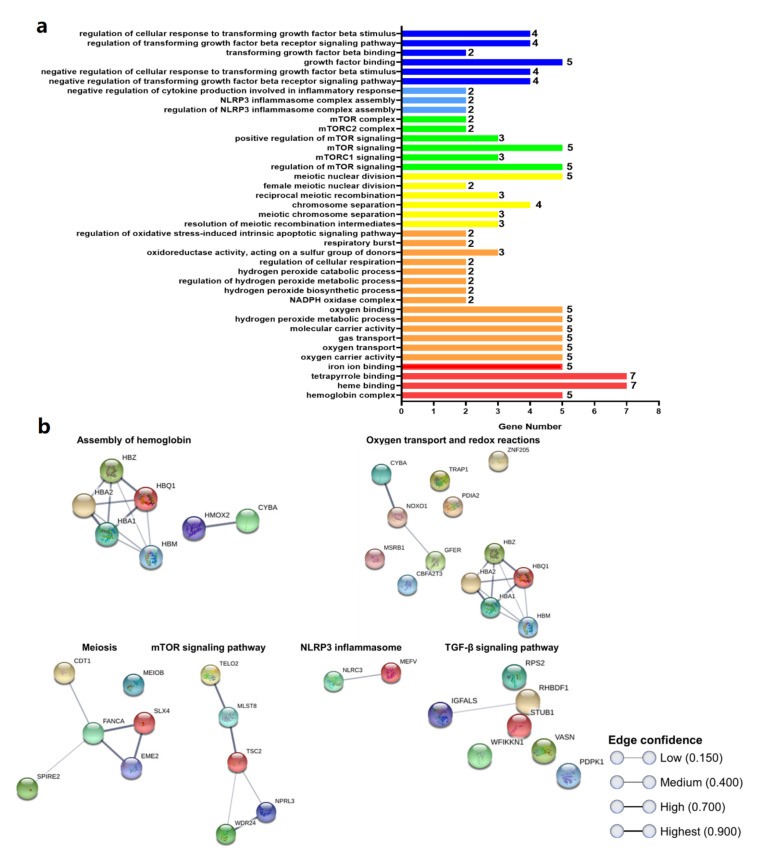
Selected signaling pathways of the six categories (**a**) and the protein–protein interaction network analysis (**b**).

According to the GO and KEGG analysis results mentioned above, the signaling pathways identified by GO analysis were divided into six major functional categories: assembly of hemoglobin, oxygen transport and redox reactions, meiosis, mTOR signaling pathway, NLRP3 inflammasome, and transforming growth factor β (TGF-β) signaling pathway (*p* < 0.05, Table S5 and Figure 7a). The PPI network analysis of these six categories was performed using String (https://string-db.org/). The identified genes and the association degrees of the proteins they encode are summarized in Figure 7b.

## 4. Discussion

The earlier and more accurate the diagnosis of abnormalities during pregnancy, the better the outcomes are likely to be for the pregnant woman and the fetus, and this is the primary goal of prenatal diagnostics. Abnormalities of chromosomal numbers and/or structure are the major causes of spontaneous abortions [16,17]. With the development of array-based molecular cytogenetic techniques and the emergence of next-generation sequencing, an increasing number of studies are using these techniques to identify the etiology and pathogenesis of numerous diseases [18,19], to improve genetic diagnosis and thus, prevention of diseases. Recently, submicroscopic CNVs have also been observed in cases of miscarriage [20,21,22] and recurrent miscarriage [23]. A recent study uncovered 44 large CNVs and three statistically significant submicroscopic CNVs (microdeletions in 22q11.21, 2q37.3 and 9p24.3p24.2), along with 309 genes primarily enriched in nervous-system development after evaluating 5180 fresh miscarriage specimens [7]. However, a clear panel of genetic markers for assessing the risk of miscarriage or RPL amongst Chinese women does not exist at present. In the present study, SNP-array and CNVs-seq were used to investigate the incidence and distribution of chromosomal abnormalities from the POCs of patients who experienced an SA or RPL. Overall, detection rates of chromosomal abnormalities in the study were 57.52% (895/1556), autosomal trisomy accounted for 64% (572/895) of cases, and chromosome 16 had the highest incidence of abnormalities, consistent with previous studies [7,24]. At the chromosomal level, the incidence of chromosomal abnormalities in RPL patients was higher than that in the SA group, but this was not related to the number of previous spontaneous abortions. With the increase in age, the incidence of chromosomal abnormalities in the SA group increased, with the lowest incidence in the individuals less than 30 years old. However, in the RPL group, chromosomal abnormalities occurred more frequently in the patients younger than 30 years old or older than 35 years old, with the lowest incidence in the 30–34 age group. The detection rate of CNVs in our study was 11.12% (173/1556), which was consistent with a previous report [25]. We noted that the detection rate of duplications (272/346) was much higher than that of deletions (2/346), which may be due to the resolution of the detection method. We also identified two recurrent RPL-associated CNVs (duplications at 16q24.3 and 16p13.3) that were significantly more common in cases of RPL compared with the SA cases group, which is a novel finding not identified in previous studies [7,26].

Chromosome 16 is 90Mb in length and contains 835 coding genes. Many of these genes have been linked to diseases, such as prenatal growth retardation [27], abnormal fetal head circumference [28], thalassemia [29] and autism [30]. The CNVs in chromosome 16 were increasingly found to serve a clear role in the determination of developmental delay [31]. Trisomy 16 is the most common cause of early miscarriage, accounting for about 6% of early miscarriages [32]. Our study results also confirmed this conclusion. The primary finding of this study was the identification of a novel duplication locus at 16q24.3 (1.65 Mb), which was found in 19 RPL cases but in none of the SA cases. Deletions in 16q24.2 have been observed frequently in patients with KBG syndrome [33,34], autism spectrum disorder, intellectual disability and congenital renal malformation [30]. Moreover, 16q24.3 alterations aggravate the clinical outcomes of head and neck squamous cell carcinoma [35]. The other RPL-associated CNV identified in this study was duplication of 16p13.3 (7.9 Mb), which was found in 18 cases of miscarriage, but not in the SA cases group. 16p13.3 has previously been identified as a novel susceptibility locus for polycystic ovary syndrome [36]. In this study, chromosomal abnormalities were most common on chromosome 16, and CNVs showed a statistically significant increase in the RPL group for the two segments of chromosome 16. Numerous genes associated with meiosis in the two CNVs were detected (such as EME2, MEIOB and SLX4). Therefore, trisomy 16 may be the result of a legacy of mutations in these genes, which cannot be detected by the CNV-seq and SNP-array methods.

Although duplications do not directly alter the entire genes in the coding regions, the modifications in the local genomic context may nevertheless shape tissue transcriptomes and thereby lead to dysregulation of the involved or neighboring genes, as reported previously [37]. Since CNVs can be very large and contain numerous genes, it is challenging to identify specific genes associated with RPL. Based on the detected CNVs, 213 genes within the two regions were identified, and functional enrichment analysis of these identified 131 signaling pathways (*p* < 0.05) was performed. These signaling pathways were classed into six major functional categories.

The physiological function of all tissues and organs depends on the maintenance of heme homeostasis. The disturbance of assembly of hemoglobin undoubtedly has a notable impact on the maintenance of normal pregnancy. There were four signaling pathways and seven genes identified in the enrichment results of the present study. Recently, reduced testicular steroidogenesis along with increased semen oxidative stress in male partners were considered as novel markers of recurrent miscarriage [38]. Previous studies have also shown that oxidative stress is associated with DNA fragmentation and leads to poor embryonic development and recurrent miscarriage [39]. In the present study, 14 signaling pathways and 13 genes were identified by enrichment analysis, and 5 of the 13 genes overlapped with the hemoglobin assembly results. One of the consequences of meiosis failure is triploidy, which is a relatively common cause of miscarriage and RPL [40]. The identification of six genes enriched in six signaling pathways in the present may thus be associated with RPL. As a master regulator of cancer, the PI3K/Akt/mTOR signaling pathway has been demonstrated previously to be involved in regulatory T cell/T helper 17 cell (Treg/Th17) differentiation [41]. The Treg/Th17 balance serves a vital role in maintaining the steady state of the maternal–fetal interface [42,43]. In addition, the activation of a reactive oxygen species–mTOR signaling axis followed by regulation of the Treg/Th17 balance was associated with recurrent spontaneous abortion [44]. There were six signaling pathways and five genes involved in the mTOR signaling pathway in the present study. Normal pregnancy requires a favorable immunological and inflammatory milieu, and a uterine hyperinflammatory state no doubt contributes to the pathogenesis of RPL. The available evidence suggests that the abnormal activation of inflammasome NLRP3 was demonstrated in the endometrium of women with unexplained RPL [45]. In the present study, three signaling pathways and two genes, MEFV and NLRC3, were identified to be of relevance based on the enrichment analysis results. The TGF-β family of cytokines, which participates in Treg cell differentiation and angiogenesis, serves important roles in the maintenance of pregnancy. Lower levels of TGF-β have been observed amongst RPL cases [46,47,48]. The enrichment analysis identified six signaling pathways and seven genes related to TGF-β in our study. Based on these results, we can speculate that duplications at 16q24.3 and 16p13.3 were two novel CNVs that might be associated with RPL.

We also noticed that there were no CNVs detected in chromosome 19. However, Wang et al. found CNVs in this chromosome in cases of miscarriage [7]; specifically the ZNF676 gene in chromosome 19 was found to be associated with recurrent miscarriage [49]. In addition, chromosome 19, which is 58.6 Mb in length and contains 1473 protein-coding genes, 918 non-coding genes, and 527 pseudogenes, has the highest gene density of any human chromosome based on ENSEMBL. As one of the first proteins synthesized by syncytiotrophoblasts, the hCG β subunit is composed of four highly homologous chorionic gonadotropin β (CGB) genes on chromosome 19, which is critical for regulating the levels of hCG, CGB5 as well as CGB8 (which is of particular importance). The association between polymorphisms in CGB genes and recurrent miscarriage has been reported previously [50,51]. Therefore, suggesting chromosome 19 is not important during the embryonic development period based on the results of this study is not accurate. Instead, chromosome 19 likely serves a very significant role during the initiation of conception and in biological evolution. We suspect that when there are microdeletions and/or microduplications on chromosome 19, the miscarriages occur sooner in the third or fourth week of gestation. There are cases of preclinical losses, such as biochemical pregnancy, and the patient may disregard it as a menstrual disorder and will thus not consult a doctor. Moreover, the lack of identification of any alterations in the present study may be due to the sample size not being large enough to detect alterations based simply on chance.

This study has several limitations. The sample size was not large enough to identify all RPL-associated CNVs. Therefore, future research requires larger cohorts with more systematic and detailed information obtained regarding the patients. In addition, high-resolution methods are required to precisely detect smaller fragments of CNVs and to identify other novel CNVs associated with RPL. The gene functional enrichment analysis performed in the present study was not systematic or in-depth, and the pathways identified are already well defined and are not considered to be of significance regarding clinical application. Further functional studies are required to develop a gene discovery approach to effectively identify candidate genes in the pathogenesis of RPL. At the same time, it is also necessary to perform basic experiments to verify these results obtained from clinical studies.

## 5. Conclusions

This study aimed to investigate the association between CNVs and the pathogenesis of RPL. Although there are some novel papers with similar results published already, only a few reports have reported the related outcomes in Chinese women. In conclusion, the results of this study showed that CNVs significantly contribute to the pathogenesis of RPL, and thus may highlight novel avenues for studies on the prevention, diagnosis and treatment of RPL, to improve live birth rates. Duplications at 16q24.3 and 16p13.3 were two novel CNVs that may be associated with RPL. A wider comprehension of the genetic mechanisms involved in RPL may lead to the establishment of a population-based diagnostic panel of genetic markers for screening for individuals at risk of RPL amongst Chinese women.

## Figures and Tables

**Table 1:** Specimen characteristics: mean maternal age and the testing results of miscarriage cases.

Times of Miscarriage	n	Frequency (%)	Age (Years old)	Normal		Abnormal	
				n	Frequency (%)	n	Frequency (%)
1	540	34.70	30.47 (range, 21–46)	262	16.84	278	17.87
2	586	37.66	31.63 (range, 21–45)	208	13.37	378	24.29
3	283	18.18	32.43 (range, 22–44)	122	7.84	161	10.35
>=4	147	9.45	33.95 (range, 24–48)	69	4.43	78	5.01
**Total**	1556	100	_	661	42.48	895	57.52

**Table 2:** Types and frequencies of chromosomal abnormality detected in products of conception in cases with different times of miscarriage using single nucleotide polymorphism-arrays and copy number variation-sequencing.

	Chromosomal Abnormality	Autosomal Trisomy		Autosomal Monosomy		Mosaicism or Triploidy		Partial Imbalance		Monosomy X		Other	
Times of Misscariage		n	Frequency (%)	n	Frequency (%)	n	Frequency (%)	n	Frequency (%)	n	Frequency (%)	n	Frequency (%)
1 (n = 278.31%)		187	21	3	0	12	1	37	4	39	4	0	0
2 (n = 378.42%)		231	26	4	0	23	3	86	10	32	4	2	0
3 (n = 161.18%)		103	12	0	0	7	1	35	4	13	1	3	0
>=4 (n = 78.9%)		51	5	1	0	4	0	15	2	7	1	0	0
Total (n = 895)		572	64	8	1	46	5	173	19	91	10	5	1

## Supplementary Materials

The following are available online at https://www.mdpi.com/2073-4425/12/2/141/s1, Table S1: CNVs detected in POCs using SNP-array and CNV-seq; Table S2: The genes in the two loci 16q24.3 and 16p13.3; Table S3: The top 10 enrichment results in three categories with *p* < 0.05 based on GO analysis; Table S4: The enrichment results of KEGG analysis; Table S5: Classification of the selected signaling pathways into 6 different categories.
